# Intraosseous schwannoma of the femur in a patient with monoclonal gammopathy of undetermined significance

**DOI:** 10.1016/j.ijscr.2020.06.054

**Published:** 2020-06-13

**Authors:** Timothy McAleese, Kevin Clesham, Darren Moloney, Andrew Hughes, Nazia Faheem, Khalid Merghani

**Affiliations:** a National University of Ireland, Galway, Co. Galway, Ireland; b Department of Trauma and Orthopaedics, Midland Regional Hospital Tullamore, Tullamore, Co. Offaly, Ireland; c Department of Histopathology, Midland Regional Hospital Tullamore, Tullamore, Co. Offaly, Ireland

**Keywords:** Intraosseous schannoma, Femoral schwannoma, Primary bone tumour, Neurilemmoma, Monocloncal gamopathy of underdetermined significance (MGUS), Case report

## Abstract

**Introduction:**

Schwannomas are slow-growing, benign tumours normally originating from the schwann cells of the nerve sheath. Intraosseous schwannomas account for 0.175% of primary bone tumours and are extremely rare, especially outside the axial skeleton. Monoclonal gammopathy has been associated with soft tissue schwannomas but never with the intraosseous variety.

**Presentation of case:**

A 55-year-old woman with a background of monoclonal gammopathy of undetermined significance (MGUS) presented with a 2-year history of right thigh pain. CT scan showed a well defined, lytic lesion with a thin peripheral rim of sclerosis in the midshaft of the femur. MRI displayed a hyperintense, well marginated and homogenous lesion. Definitive diagnosis was made based on the classical histopathological appearance of schwannoma. We managed our patient with local curettage and prophylactic cephalomedullary nailing based on her high mirel score.

**Discussion:**

Intraosseous schwannomas are poorly understood but most commonly reported in middle-aged women. Radiologically, their differential diagnosis includes malignant bone tumours, solitary bone cysts, aneurysmal bone cysts and giant cell tumours. As a result, they are usually diagnosed incidentally on histology. Although malignant transformation is possible in soft tissue schwannomas, all intraosseous schwannomas reported to date have been benign.

**Conclusion:**

This case demonstrates the importance of suspecting intraosseous schwannoma as a differential diagnosis for lytic bone lesions to avoid the overtreatment of patients. We also highlight monoclonal gammopathy of undetermined significance as a potential risk factor for a poorly understood disease and make recommendations about the appropriate management of these lesions.

## Highlights

• Intraosseous schwannoma are a rare but important lytic bone lesion on imaging.
• There may be an association between primary intraosseous schwannoma and monoclonal gammopathy of undetermined significance (MGUS).
• Definitive diagnosis of intraosseous schwannoma is based on a classical histological appearance.
• Intraosseous schwannoma is benign and can be treated by local surgical excision alone including in the setting of MGUS.

## 1 Introduction

Intraosseous schwannoma is a rare condition accounting for 0.175% of primary bone tumours [1]. Furthermore, long bone involvement of these tumours is unusual and the majority of intraosseous schwannomas are found in the skull, mandible and spine [[1], [2], [3]]. As a result, they are often misdiagnosed for more serious lytic lesions of bone including metastatic disease and osteomyelitis, leading to the over-treatment or over-investigation of patients [4]. We also highlight monoclonal gammopathy of unknown significance (MGUS) as a potential risk factor for developing primary intraosseous schwannoma. MGUS is a plasma cell dyscrasia that leads to bone marrow and blood infiltration with monoclonal antibodies or M protein. Our case report helps further characterise the common sites, patient demographics and risk factors associated with primary intraosseous schwannoma of the femur, that remain undefined [[5], [6], [7], [8], [9]].

The case report satisfies the SCARE criteria of reporting [10].

## 2 Case Presentation

**Fig. 1:**
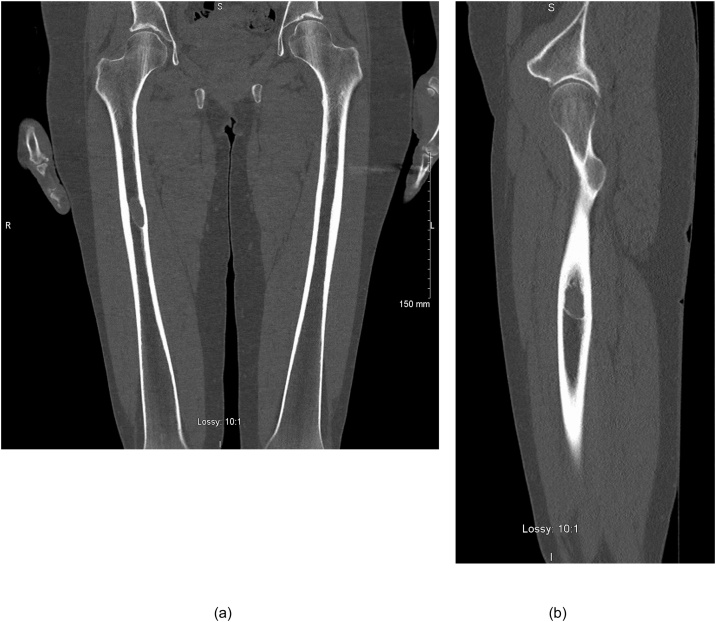
***(a,b) Coronal and Saggital sections from CT Skeletal survey for paraproteinaemia***. Single 3.2 × 1.5 cm medium low attenuation lesion with a thin sclerotic margin in the midshaft of the right femur causing mild scalloping of the adjacent inner aspect of the medial cortex.

A 55-year-old woman was referred to the orthopaedic department with a 2-year history of mild right thigh pain exacerbated by long walks or running and a CT scan showing a well-defined lesion in the midshaft of her right femur. She was initially seen by the haematology services when she was diagnosed with neutropaenia (0.9 × 10^9^/L) following a viral illness. Further immunological assay testing measured high IgG levels and subsequent serum protein electrophoresis revealed two clonal bands, band 1 IgG 15.5 g/l and band 2 IgA kappa 5.2 g/l (including beta 2 band). These paraprotein levels have remained stable over 12 months. Her 24-hr urine light chain assay was normal. Assessment of plasma cell infiltration of the bone marrow by biopsy was deemed unnecessary at this time as our patient was diagnosed with IgG type monoclonal gammopathy of undetermined significance (MGUS) with a stable paraprotein level. However, CT skeletal survey was ordered as part of the work-up for her thigh pain and showed the lesion in the midshaft of the right femur (Fig. 1).

On exam, the femur appeared normal, the overlying skin was intact and there was no warmth, erythema or induration. The right hip and knee had normal movement with a full range of motion. There was no evidence of lymphadenopathy in the groin or popliteal fossa. Her only contributary past medical history was IgG MGUS. Her family history includes a sister with congenital hypothyroidism and a brother who passed away aged 18 from neuroblastoma of the kidney.

Her initial laboratory investigations showed: Total WCC 2.7, Neutrophils 0.9–1.2 × 10^9^/L, monocyte count 0.19, Platelet count 283 × 10^9^/L, ESR 37. CRP 1. Urea, Creatinine, Electrolytes, TFT, B12, Folate, Ferritin were all within normal parameters. Subsequent FBC at 3 months follow-up showed her neutrophil level had returned to normal ranges. LDH (186 u/l) was also normal at this time.

**Fig. 2:**
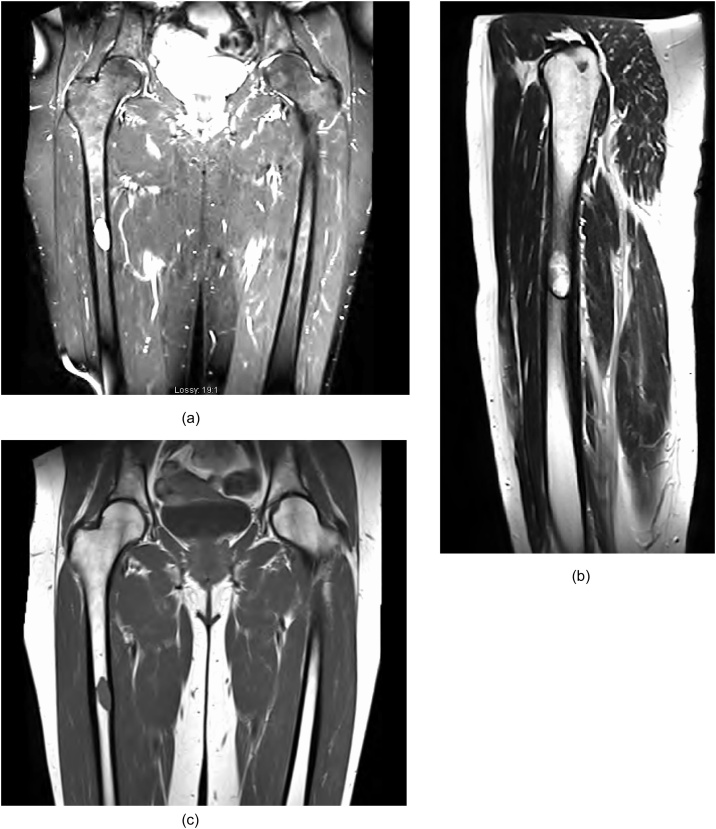
***MRI with standard multi-planar technique***. (a, b) Coronal and sagittal T2 weighted MRI images of a 1.4 × 2.0 × 3.3 cm showed a hyperintense, well marginated, eccentrically located and marginally expansile lesion involving the mid right femoral shaft. (c) Coronal T1-weighted image showed a well circumscribed cystic lesion of the right femoral shaft with a narrow zone of transition. It was fluid filled and has a relatively homogenous consistency with no cortical expansion. No other focal lesion seen.

CT demonstrated evidence of a lytic lesion with surrounding sclerosis eroding the cortex of the femoral diaphysis (Fig. 1). Subsequent magnetic resonance imaging detailed a well marginated, low T1 and high T2 signal, eccentrically located and marginally expansile lesion with a homogeneous consistency (Fig. 2).

**Fig. 3:**
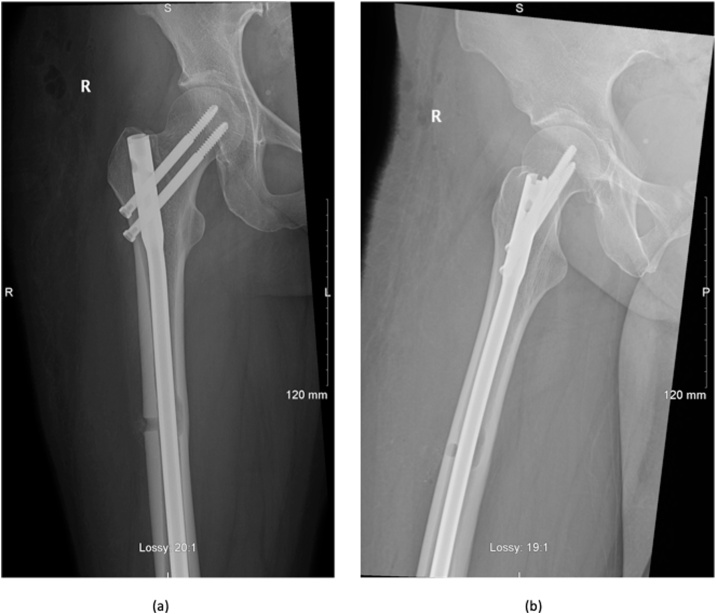
***(a,b)* Femur XR post-operatively.** The Intramedullary nail in situ in satisfactory position with evidence of excision biopsy site and cortical erosion.

She underwent simultaneous excision biopsy of the lesion and prophylactic cephalo-medullary nailing based on her Mirel score of 8. Her operation was successfully performed under consultant supervision and she was discharged day 4 post-operatively after receiving care from the inpatient physiotherapy department (Fig. 3).

**Fig. 4:**
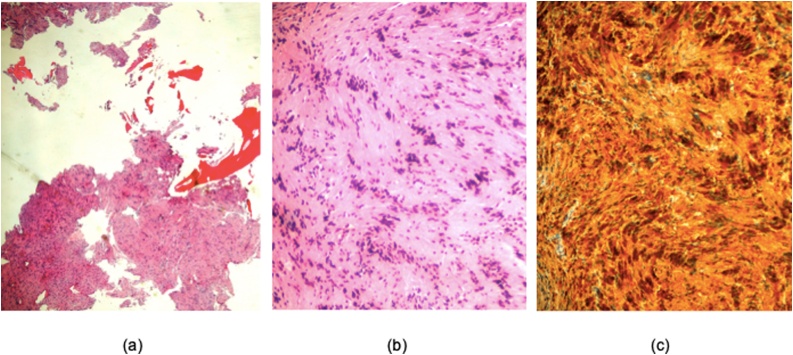
(a) Low power image showed a fragmented spindle cell lesion and accellular necrotic bone. (b) High power image showed biphasic appearance with hypercellular Antoni A areas and myxoid hypocellular Antoni B areas. There was evidence of nuclear pallisading around fibrillary processes (verocay bodies) and variable cellular spindle cell proliferation consistent with schwannoma. No atypical infiltrate or evidence of malignancy was seen. (c) Cytoplasmic and nuclear immunohistochemical staining demontrated that the neoplastic cells are positive for S100.

Histology with immunochemistry results exhibited variable cellular spindle cell proliferation and with verocay bodies consistent with schwannoma. No atypical infiltrate or evidence of malignancy was seen. Immunohistochemical staining detected that the lesional cells were positive for S100 and negative for SMA and Desmin (Fig. 4).

Our patient has recovered full mobility after an uncomplicated post-operative period. She has been discharged from orthopaedic follow-up after 3 months. She attends the haematology annually to ensure her paraprotein levels remain stable.

## 3 Discussion

Generally, schwannomas affect the outer sheath of nerve cells and therefore occurs in soft tissues. Intraosseous schwannoma accounts for 0.175% of primary bone tumours and is most common in the axial skeleton especially the skull, spine and mandible. This is thought to be due to the density of sensory nerves in these regions [[1], [2], [3]]. They seem to have 2:1 predominance for females and are found most commonly in patients aged between 20–50 but can occur at any age including paediatric populations [4]. The process by which schwannomas develop in bone is poorly understood but there are 3 main patterns described. The most widely accepted are that they arise from either the nerves in the nutrient foramina entering the bones forming a dumbbell-shaped tumour or that they form within the medullary cavity from the non-myelinated nerves associated with blood vessels. They may also be extra-osseous and cause secondary erosion of the bone [1,5,9].

The majority of primary intraosseous schwannomas are sporadic lesions but they have been found associated with Carney syndrome and neurofibromatosis 1 (von Recklinghausen’s disease) [1,11]. Soft tissue schwannomas and monoclonal gammopathy have been previously associated [12,13]. Daniel et al. also report an association of unknown significance between tibial nerve schwannoma and multiple myeloma, a disease that develops from the progression of MGUS [14]. MGUS is known to be associated with a form of peripheral neuropathy known as distal acquired demyelinating symmetric neuropathy (DADS-M). This is inflammatory in nature and may contribute to the pathogenesis of schwannoma [15]. Immunohistochemically, MGUS (in 10%) and Schwannoma are CD 56 positive. Although there is a lack of clear insight regarding the control mechanisms for oncogenesis or the relationship between both diseases. There have been several associations mentioned making it less likely to be purely coincidental.

Our case demonstrates the classical radiologic findings in intraosseous schwannoma including a well-defined osteolytic lesion with sclerotic borders; a lobulated or trabeculated contour; cortical erosions; and absence of central calcification. It is important to note that this appearance can be difficult to differentiate from severe diagnosis such as malignancy or osteomyelitis [5]. Furthermore, they are difficult to distinguish from other differential diagnoses such as aneurysmal bone cyst, solitary bone cysts, benign chondroblastomas, giant cell tumours and chondromyxoid fibroma [16]. Diagnosis of schwannoma in the soft tissue is often made by the MRI appearance alone. Intraosseous schwannoma displays similar MR characteristics. They are isointense to skeletal muscle on T1-weighted imaging and hyperintense on T2-weighted imaging. Heterogeneous and homogenous appearances of intra-osseous schwannoma have been described in the literature [[5], [6], [7]]. Schwannoma can also demonstrate the “string sign” or an attenuated margin above and below the lesion [17].

As these tumours are cliniradiologically indistinguisable from their differential diagnoses and have an extremely low incidence they are typically diagnosed histopathologically. Microscopically, they have similar appearances to soft tissue schwannoma and demonstrate two types of cell arrangements, Antoni A and Antoni B. Type A tissue is composed of densely packed, spindle-shaped cells arranged in bundles and cords surrounded by an eosinophilic cytoplasm. The nuclei of these cells are often arranged in palisading rows, forming Verocay bodies. Type B tissue has loosely scattered, pleomorphic schwann cells with predominantly myxoid cytoplasm. They have thick-walled and hyalinised blood vessels. Microcystic areas with haemorrhage are common. Diffuse immunoreactivity for S100 protein is indicative of schwann cell origin. Negative staining for smooth muscle markers such as desmin and smooth muscle actin (SMA) are used to rule out histological differential diagnoses [[6], [7], [8], [9],^16^]. The subtle difference between intraosseous schwannoma and soft tissue schwannoma is the higher degree of cellularity with subtle palisading and poorly formed verocay bodies [16].

Although malignant transformation is possible in soft tissue schwannomas, all intraosseous schwannomas reported to date have been benign [18]. There are however reports of local recurrence. Wirth et al. reviewed 31 cases and found a 16% recurrence rate in those who had incomplete removal while recurrence was not seen after complete resection [19]. The current literature supports treatment by complete curettage or marginal excision, with or without bone grafting [20,21]. In our patient, we elected for prophylactic cepahlomedullary nailing of the femur based on a Mirel score of 8 for mild pain and a lytic lesion with a size of 1/3 to 2/3 of the bone’s diameter.

## 4 Conclusion

Intraosseous schwannoma is an extremely rare diagnosis, especially in the long bones. It can be difficult to differentiate from other radiographically benign-looking, lytic bone lesions but is an important differential to consider in patients with chronic, insidious thigh pain. MGUS may represent a potential risk factor for intraosseous schwannoma. Finally, although malignant transformation has been reported in schwannomas of soft tissues, all intraosseous schwannomas reported to date have been benign, such that curettage or marginal excision remains the therapy of choice.

## Sources of funding for your research

This research did not receive any specific grant from funding agencies in the public, commercial, or not-for-profit sectors.

## Ethical approval

Ethical approval was not required and patient-identifying information was not presented in this report.

## Consent

Written informed consent was obtained from the patient for publication of this case report and accompanying images. A copy of the written consent is available for review by the Editor-in-Chief of this journal on request.

## Author contribution

**Timothy McAleese:** Conceptualization, Data curation, Methodology, Project administration, Software, Writing - original draft.

**Kevin Clesham:** Conceptualization and design, Project administration, Writing - review & editing.

**Darren Moloney:** Conceptualization, Formal analysis, Investigation, Writing - review & editing.

**Andrew Hughes:** Conceptualization, Administration, Validation, Visualization, Writing - review & editing.

**Nazia Faheem:** Conceptualization, Administration, Resources, Software, Formal analysis, Writing - review & editing.

**Khalid Merghani:** Supervision, Conceptualization, Validation, Writing - review and editing.

All authors read and approved the final manuscript.

## Registration of research studies

This is not applicable due to the nature of this case report.

## Guarantor

Mr Khalid Merghani is a consultant orthopaedic surgeon and the senior author in this paper. As guarantor he is responsible for the work and/or the conduct of the study, had access to the data and controlled the decision to publish.

## Provenance and peer review

Not commissioned, externally peer-reviewed.

## Declaration of Competing Interest

The authors have no conflicts of interest to declare.

## References

[1] Fawcett K.J., Dahlin D.C. (1967). Neurilemmoma of bone. Am. J. Clin. Pathol..

[2] Mutema G.K., Sorger J. (2002). Intraosseous schwannoma of the humerus. Skeletal Radiol..

[3] Isaac J., Shyamkumar N.K., Karnik S.V. (2004). Intraosseus schwannoma. J. Postgrad. Med..

[4] Ida C.M., Scheithauer B.W., Yapicier O., Carney J.A., Wenger D.E., Inwards C.Y. (2011). Primary schwannoma of the bone: a clinicopathologic and radiologic study of 17 cases. Am. J. Surg. Pathol..

[5] Wang X.J., Hartley K., Holt G.E., Fadare O., Cates J.M. (2014). Intracortical schwannoma of the femur. Skeletal Radiol..

[6] Verma R.R., Khan M.T., Davies A.M., Mangham D.C., Grimer R.J. (2002). Subperiosteal schwannomas of the femur. Skeletal Radiol..

[7] Hoshi M., Takada J., Oebisu N., Nakamura H. (2012). Intraosseous schwannoma of the proximal femur. Asia. J. Clin. Oncol..

[8] Sanado L., Ruiz J.L., Laidler L., Polo M. (1991). Femoral intraosseous neurilemoma. Arch. Orthop. Trauma Surg..

[9] Perera N., de Silva C., Perera V. (2017). Large schwannoma of the femur - a common tumor at an unusual site: a case report and review of the literature. J. Med. Case Rep..

[10] Agha R.A., Borrelli M.R., Farwana R., Koshy K., Fowler A.J., Orgill D.P. (2018). The SCARE 2018 statement: updating consensus surgical CAse REport (SCARE) guidelines. Int. J. Surg..

[11] Kashima T.G., Gibbons M.R., Whitwell D., Gibbons C.L., Bradley K.M., Ostlere S.J. (2013). Intraosseous schwannoma in schwannomatosis. Skeletal Radiol..

[12] Damasena I., Low I., Carey-Smith R. (2013). Retroperitoneal schwannoma with monoclonal plasma cell infiltration: an exceptionally rare collision tumor?. Int. J. Surg. Pathol..

[13] Oda K., Miyasaki K., Ohta M., Shibasaki H. (1987). A case of myasthenia gravis associated with thymoma, multiple schwannomas and monoclonal IgA gammopathy. Jpn. J. Med..

[14] Daniel M., Waters D., Chen C., Brouyette N. (2019). Posterior tibial nerve schwannoma in a multiple myeloma patient: a case report. SAGE Open Med. Case Rep..

[15] Ahn A., Park C.J., Cho Y.U., Jang S., Seo E.J., Lee J.H. (2020). Clinical, laboratory, and bone marrow findings of 31 patients with waldenstrom macroglobulinemia. Ann. Lab. Med..

[16] de la Monte S.M., Dorfman H.D., Chandra R., Malawer M. (1984). Intraosseous schwannoma: histologic features, ultrastructure, and review of the literature. Hum. Pathol..

[17] Dunnick Nr. (2000). Image interpretation session: 1999. Intraosseous malignant peripheral nerve sheath tumor (malignant schwannoma) in a patient with neurofibromatosis. Radiographics.

[18] Jacobs R.L., Fox T.A. (1972). Neurilemoma of bone. A case report with a review of the literature. Clin. Orthop. Relat. Res..

[19] Wirth W.A., Bray C.B. (1977). Intra-osseous neurilemoma. Case report and review of thirty-one cases from the literature. J. Bone Joint Surg. Am..

[20] Palocaren T., Walter N.M., Madhuri V., Gibikote S. (2008). Schwannoma of the fibula. J. Bone Joint Surg. Br..

[21] Ang W.M., Yates P., Robbins P., Wood D. (2008). Recurrent benign solitary intraosseous schwannoma of the tibia. Orthopedics.

